# Editorial to the Special Issue–“Natural Products and Drug Discovery”

**DOI:** 10.3390/molecules25051128

**Published:** 2020-03-03

**Authors:** Pinarosa Avato

**Affiliations:** Dipartimento di Farmacia-Scienze del Farmaco, Università degli Studi di Bari Aldo Moro, Via Orabona 4, 70125 Bari, Italy; pinarosa.avato@uniba.it

Natural products hold a prominent position in the discovery and development of many drugs used nowadays, with diverse indications for human and animal health. Especially, plants have played a leading role as source of specialized metabolites with medical effects, while other organisms such as marine and terrestrial animals and microorganisms produce very important drug candidate molecules. Specialized metabolites from all these natural sources can be used directly as bioactive compounds, or as drug precursors. Due to their wide chemical diversity they can act as drug prototypes and/or be used as pharmacological tools for different targets.

Many scientists have contributed to this Special Issue-SI which includes 21 papers, among them original articles as well as survey articles, that give the readers of *Molecules* updated and new perspectives about natural products in drug discovery.

Trabace et al. [1] reported the impact of celastrol, a pentacyclic terpene produced by the medicinal plant *Tripterygium wilfordii*, on behavioural dysfunctions observed in adult mice exposed to subanesthetic doses of the N-methyl-D-aspartate receptor antagonist ketamine at PNDs. This study suggested that the NOX inhibition by the early administration of celastrol can prevent ketamine-induced psychotic-like behavioural dysfunctions, as well as the increase of cerebellar oxidative stress and the reduction of anti-inflammatory cytokines. Results open up new pharmacological insights into the possible use of this phytochemical for neuroprotection during brain development.

The current production of artemisinin, the antimalarial drug from *Artemisia annua*, is still mainly based on the use of cultivated plants. Various alternative strategies have been explored to improve its production in the plant. In their paper, Simonsen et al. [2] describe the heterologous expression of artemisinin biosynthetic pathway in *Physcomitrella patens*, showing novel insights into the potential of this model organism for artemisinin production. The moss was shown to express endogenous enzymes with similar activity to that for artemisinin biosynthesis in *A. annua*, suggesting the possibility of engineering artemisinin biosynthesis and that of other related high-value terpenoids in *P. patens*.

Recent data highlight that glucosinolates, the sulfur compounds produced in the Brassicaceae plant family, have pain-relieving efficacy. Mannelli and coworkers [3] describe the anti-hyperalgesic efficacy of a defatted seed meal of *Eruca sativa* along with glucoerucin, its main glucosinolate, on streptozotocin induced diabetic neuropathic pain in mice. Both myrosinase bio-activated *E. sativa* meal and glucoerucin showed a dose-dependent pain relief effect in diabetic mice, with the meal being more active. Co-administration of the meal and glucoerucin with H_2_S scavengers abolished the induced pain relief. The authors also showed that repeated treatments did not induce tolerance to the anti-hypersensitive effect. The paper nicely indicates a potential of *E. sativa* seed meal to treat patients with diabetic neuropathy.

Wolfender et al. [4] propose a new approach to discover new bioactive natural products. They make use of a metabolomic strategy in combination with multivariate data analysis and multi-informative molecular maps to profile extracts of *Bacopa* species (*B. monnieri, B. caroliniana,* and *B. floribunda*) and screen for anti-lipid peroxidation activity. This approach allowed the identification of six inhibitors of lipid peroxidation from the three *Bacopa* species. Three of them were novel molecules. Data obtained by this method permitted to discover the potential bioactivity for each compound directly from the crude plant extracts prior to any physical separation process.

The genus *Phyllantus* includes some Cuban endemic species traditionally used for the treatment of different diseases. Wessjohann et al. [5] report on the chemical characterization of the aqueous extract from *Phyllantus,* known to have in vitro antiviral activity and protective effect against UV-light induced DNA damage and genotoxicity. The chemical structure of a novel *C*-glycosylated flavonol, named fideloside, with a promising anti-inflammatory capacity in human explanted monocytes is described.

Tran et al. [6] describe the anti-tumor effect of 2′,4′-dihydroxy-6′-methoxy-3′,5′- dimethylchalcone (DMC) against some human pancreatic cancer cell lines. In a cell proliferation assay, the compound, isolated from buds of *Cleistocalyx operculatus,* was shown to be cytotoxic against PANC-1 and MIA PACA2 cells in a dose-dependent manner. In addition, treatment with DMC led to apoptosis of PANC-1 cells inducing proteolytic activation of caspase-3. A possible use of this natural product as chemotherapeutic agent to fight human pancreatic cancer is suggested.

The paper by Avato and Argentieri [7] describes for the first time the chemical profile of a commercial spagyric tincture prepared from the dried roots of devil’s claw. Compositional consistence of this preparation over time was investigated by comparison with an already expired devil’s claw spagyric tincture from the same producer. The two preparations had no significant compositional variations. In addition, their antioxidant potential based on the DPPH assay showed similar IC50. From this investigation, it could be demonstrated that the two spgayric tinctures maintain good stability and biological activity for at least four years after production.

While several species of *Astragalus* have been extensively investigated, phytochemical and pharmacological information on *A. boeticus* is very limited. Scognamiglio et al. [8] report on the chemical characterization of acylatd cycloartane glycosides from this species and on their cytoxicity towards human colorectal cancer cell lines. The authors show that, among the five isolated cycloartane-type glycosides, 6-*O*-acetyl-3-*O*-β-d-xylopyranosylcycloastragenol, with acylation at C-3 and C-6 and the C-25 free hydroxyl function, had the highest activity, thus confirming some structural requirements for the cytotoxicity of cycloartane derivatives.

The study by Rivière et al. [9] reports on the antimicrobial effect of hop extracts and their main prenylated phenolics (xanthoumol, desmethylxanthohumol and lupulone) against MRSA strains, and on their antiparasitic activity against *Trypanosoma brucei* and *Leishmania mexicana.* Besides considering the antibacterial effect of single hop components, the authors also describe the positive effect obtained by different combinations of xanthoumol with desmethylxanthohumol or with lupulone. They also investigated post-antibiotic effects and found that xanthoumol and desmethylxanthohumol cause a significant delay of bacterial re-growth. Among hops active principles, lupulone was shown as the most active against *T. brucei* and *L. mexicana,* while humulone was the less active.

The paper by Koh et al. [10] represents an extensive phytochemical investigation of *Lee indica,* an evergreen perennial shrub/small tree distributed in Southeast Asia, traditionally used as medicinal plant with various indications. A total of 31 compounds belonging to different chemical groups (flavonoids, coumarins, oxylipins, etc.) have been identified. Three of them are novel dihydrochalcones: 4′,6′-dihydroxy-4-methoxydihydrochalcone 2′-*O*-rutinoside, 4′,6′-dihydroxy-4-methoxydihydro chalcone 2′-*O*-glucosylpentoside and 4′,6′-dihydroxy-4-methoxydihydrochalcone 2′-*O*-(3″-*O*-galloyl)-β-D-glucopyranoside.

Tava et al. [11] illustrate the chemical and biological diversity in terms of phenolics content and antioxidant capacity of leaves and flowers extracts from a set of *Trifolium* species originating from contrasting growing environments. Variations in the distribution of total phenolics were found between lowland and mountain germplasm rising some considerations on the different adaptive strategies. Accordingly, differences in the scavenging capacity of clove extracts from lowland and mountain germplasm and/or plant part were observed. Based on these results, the authors also discuss the possible link between environmental factors, chemical composition, and content of phenolics in *Trifolium.*

Marine organisms are an important resource of peptides which due to their unique structure may have several physiological functions. Pang et al. [12] designed the synthesis of seven new tripeptide derivatives of the marine cyclopeptide xyloallenoide A to investigate their capacity to promote cellular proliferation in human endotelial cells and zebrafish embryos. With their study, the authors gain interesting Structural-Activity-Relationships. For example, it was shown that tripeptides containing L-Tyr or D-Pro fragments have a higher potency to promote the cellular proliferation of human endotelial cells.

The hypoglycemic effect and the mechanism of action of an ethanolic extract of *Sennae folium* on L6 rat skeletal muscle cells are described by Yang et al. [13]. The drug shows a strong effect in promoting glucose uptake, GLUT4 expression and translocation and promotes cytosolic Ca^2+^ levels. It is thus suggested that *Sennae folium* might have a role for the treatment of insulin resistance.

Hartmann et al. [14] describe the composition of sulfated metabolites from the siphonous green alga *Dasycladus vermicularis*, widely distributed throughout tropical to temperate regions. The phytochemical analysis led to the isolation of two sulfated phenolic acids and four sulfated coumarins including two novel compounds, 5,8′-di-(6(6′),7(7′)-tetrahydroxy-3-sulfoxy-3′-sulfoxycoumarin), named dasycladin A and 7-hydroxycoumarin-3,6-disulfate, named dasycladin B. In addition, for the first time, a validated HPLC method for the separation and quantification of sulfated coumarins is presented.

The work by Sulaiman et al. [15] aims to study the analgesic effect of cardamonin, isolated from *Boesenbergia rotunda.* Its antinociceptive activity was examined using chemical and thermal mice models of nociception. The authors show that cardamonin is able to produce significant analgesia in formalin-, capsaicin- and glutamate-induced paw licking tests. In addition, they demonstrated that the phytochemical induces a significant increase in the response latency time of animals subjected to hot-plate thermal stimuli. In conclusion, this study shows that cardamonin exerts significant peripheral and central antinociception in mice through the involvement of TRPV1, glutamate and opioid receptors.

Ho et al. [16] investigated the chemical profile of *Imperata cylindrica* and the growth inhibitory effects of each identified constituent on different cancer cell lines. They achieved the isolation of 2-methoxysterone, 11,16-dihydroxypregn-4-ene-3,20-dione, and tricin, which were found to inhibit the growth of some breast and colon cancer cell lines.

The study by Chang et al. [17] aimed to further characterize the cytotoxic constituents from *Dryopteris fragrans,* a valuable medicinal plant with anti-cancer activity. Isolation of six known compounds plus two new bioactive phenolics, namely dryofragone and dryofracoumarin B, was achieved by a cytotoxicity-guided tracking. The immunomodulatory capacity of these compounds has also been described and results showed that some of them may activate the LPS signaling pathway thus affecting the growth of tumor cells through immuno-regulation.

Lai et al. [18] review the activity of natural terpenes and their derivatives against pathogenic bacteria with particular attention to terpenes effective in the treatment of microbial resistance. They also discuss future prospects, such as new natural sources, drug delivery systems to be used in clinical trials, possible structural modification, either synthetically or via biotransformation, to increase the bioactivity, and the development of combination drugs with fewer side effects.

Marine organisms are a potential sustainable source of peptides that act as ACE inhibitors and are considered as therapeutic agent to combat hypertension. The review by Pujiastuti et al. [19] summarizes information on their distribution among marine organisms, their production, chemical characterization, and bioactivity.

The role of carotenoids to counteract oxidative stress and promote healthy aging is discussed by Tan and Norhalzan [20]. As many studies have shown an inverse relationship between carotenoids and age-related diseases by reducing oxidative stress, carotenoids are potential candidates to counteract age-associated pathologies. Besides a description of the chemical types of carotenoids and their natural sources, the authors review the underlying mechanisms of action to understand their role on human health.

The review by Scharenberg and Zidorn [21] illustrates the phytochemistry of the holoparasitic genus *Orobanche.* Both genuine metabolites produced by *Orobanche* species as well as natural products sequestered from their host plants are reviewed. In addition, an overview of the biological activity of extracts and pure compounds from different species of *Orobanche* is also given. Information was retrieved from SciFinder and ISI Web of Knowledge databases, taking into account reports until the end of 2017.

Overall, this special issue contributes to highlighting new biological activities for known plants and natural compounds. In addition, some of the studies have disclosed novel plant molecules with promising pharmacological applications.
